# Necrosis of Inferior Maxilla, Involving the Whole Bone

**Published:** 1862-06

**Authors:** 


### Selections.

Necrosis of Inferior Maxilla involving the whole Bone.—There are two causes for the necrosis in this case, viz., the fumes of phosphorus, while working in a match factory, and a cold contracted while living in a damp cellar, after having taken calomel. Great enlargement of the lower part of the face is a peculiar feature of necrosis, resulting from exposure to the fumes of phosphorus; this kind of necrosis is *sui generis*. The treatment of this case should be directed primarily to the general health—the diseased parts should be washed with a solution of chlorate of potash, two or three times a day; afterward the openings should be enlarged, and the dead bone extracted. It is not a difficult matter to remove a very large piece of the jaw-bone, but that is an operation entirely distinct from the removal of necrosed bone in such a case as this. This girl was in a match factory three years, exposed to the fumes of phosphorus, which has produced its peculiar effect, and resembles mercury in that both generally attack the alveoli first; in this case the alveoli seem to be sound. The true way to proceed, in removing the sequestrum, is to enlarge the sinuses, and perhaps connect one or more so as to insert the fingers, and feel for the size and direction of diseased bone; after the condition is thoroughly ascertained, the sequestrum may be seized, and generally pulled out by a sufficient amount of patience and perseverance. This is an entirely distinct business from removing a portion of jaw-bone.

There are other forms of disease of the jaw-bone, among which are the malignant and cystic. The latter form was first discovered and described by a French surgeon named Forget, about eleven years ago. It is produced by an abnormal growth of the molar teeth, which may come crosswise, inverted, etc., producing cysts, and ultimate disease of bone. This form of disease is not malignant. The professor has obtained his principal knowledge of this disease from the study of cases which have come into the hands of dentists. The proper remedy is the extraction of the offending tooth.—*Med. and Surg. Reporter.*

